# A deep learning-based application for COVID-19 diagnosis on CT: The Imaging COVID-19 AI initiative

**DOI:** 10.1371/journal.pone.0285121

**Published:** 2023-05-02

**Authors:** Laurens Topff, José Sánchez-García, Rafael López-González, Ana Jiménez Pastor, Jacob J. Visser, Merel Huisman, Julien Guiot, Regina G. H. Beets-Tan, Angel Alberich-Bayarri, Almudena Fuster-Matanzo, Erik R. Ranschaert, on behalf of the Imaging COVID-19 AI initiative

**Affiliations:** 1 Department of Radiology, Netherlands Cancer Institute, Amsterdam, The Netherlands; 2 GROW School for Oncology and Reproduction, Maastricht University, Maastricht, The Netherlands; 3 Quantitative Imaging Biomarkers in Medicine (Quibim), Valencia, Spain; 4 Department of Radiology and Nuclear Medicine, Erasmus MC, University Medical Center Rotterdam, Rotterdam, The Netherlands; 5 Department of Radiology and Nuclear Medicine, Radboud University Medical Center, Nijmegen, The Netherlands; 6 Department of Pneumology, University Hospital of Liège (CHU Liège), Liège, Belgium; 7 Department of Radiology, St. Nikolaus Hospital, Eupen, Belgium; 8 Ghent University, Ghent, Belgium; Fatebenefratelli Isola Tiberina - Gemelli Isola, ITALY

## Abstract

**Background:**

Recently, artificial intelligence (AI)-based applications for chest imaging have emerged as potential tools to assist clinicians in the diagnosis and management of patients with coronavirus disease 2019 (COVID-19).

**Objectives:**

To develop a deep learning-based clinical decision support system for automatic diagnosis of COVID-19 on chest CT scans. Secondarily, to develop a complementary segmentation tool to assess the extent of lung involvement and measure disease severity.

**Methods:**

The Imaging COVID-19 AI initiative was formed to conduct a retrospective multicentre cohort study including 20 institutions from seven different European countries. Patients with suspected or known COVID-19 who underwent a chest CT were included. The dataset was split on the institution-level to allow external evaluation. Data annotation was performed by 34 radiologists/radiology residents and included quality control measures. A multi-class classification model was created using a custom 3D convolutional neural network. For the segmentation task, a UNET-like architecture with a backbone Residual Network (ResNet-34) was selected.

**Results:**

A total of 2,802 CT scans were included (2,667 unique patients, mean [standard deviation] age = 64.6 [16.2] years, male/female ratio 1.3:1). The distribution of classes (COVID-19/Other type of pulmonary infection/No imaging signs of infection) was 1,490 (53.2%), 402 (14.3%), and 910 (32.5%), respectively. On the external test dataset, the diagnostic multiclassification model yielded high micro-average and macro-average AUC values (0.93 and 0.91, respectively). The model provided the likelihood of COVID-19 vs other cases with a sensitivity of 87% and a specificity of 94%. The segmentation performance was moderate with Dice similarity coefficient (DSC) of 0.59. An imaging analysis pipeline was developed that returned a quantitative report to the user.

**Conclusion:**

We developed a deep learning-based clinical decision support system that could become an efficient concurrent reading tool to assist clinicians, utilising a newly created European dataset including more than 2,800 CT scans.

## Introduction

Coronavirus disease 2019 (COVID-19), caused by severe acute respiratory syndrome coronavirus 2 (SARS-CoV-2), has become a global health emergency since its appearance by the end of 2019 [1]. Dyspnoea, fever, dry cough, and myalgia are common manifestations of COVID-19. However, its clinical presentation is variable, ranging from asymptomatic to severe and potentially fatal [2]. As a result, COVID-19 continues to present challenges in diagnosis and patient monitoring.

At present, the reference standard for diagnosis is the reverse transcriptase polymerase chain reaction (RT-PCR) test [3]. However, this technique has known limitations due to variations in sensitivity and longer turnaround times in certain settings which can affect key decisions in clinical routine. Additionally, the limited availability of RT-PCR and the lack of experienced personnel to run the analysis may be a problem in some countries [4, 5].

Several studies have focused on the potential use of chest imaging for diagnostic purposes. Although chest imaging is not indicated for routine screening of asymptomatic individuals or patients with mild symptoms [6], it is able to identify alternative causes of respiratory symptoms, such as bacterial infection, and may help to define disease stage, assess disease progression and improve prognostication in symptomatic COVID-19 patients [7, 8]. Computed tomography (CT) is the gold standard imaging modality for the diagnosis of COVID-19 pneumonia [7], and seems to have great diagnostic and prognostic value for COVID-19 as evidenced in several studies [9–16]. Thus, some studies even showed a higher sensitivity than RT-PCR in diagnosing COVID-19 [9–12], allowing to detect ground-glass opacities—lung lesions commonly found on CT in COVID-19 patients—[9, 13, 14] and even abnormalities and changes over time in asymptomatic patients [15, 16]. The fast turnaround time of CT imaging is also worth mentioning [17, 18]. Nevertheless, as discussed by Mair et al. [12], CT specificity remains low, which makes it unlikely to replace PCR as the gold standard test. The advantages already discussed, however, suggest that a CT imaging-based decision support tool could improve patient’s management, becoming a useful complementary test to RT-PCR or even alternative in special circumstances (e.g., in cases of work overload or resource shortage). Additionally, there may be a role in the opportunistic screening of COVID-19 on routinely performed chest CT scans in different settings, which could be especially relevant for oncologic patients.

Interestingly, deep learning-based applications have the potential to optimise image interpretation, by improving performance in detection, characterisation and quantification tasks, especially in the field of chest imaging [19]. They also facilitate the automatization of processes, reducing the diagnostic inconsistencies from inter- and intra-reader diagnoses. Therefore, deep learning models based on CT scans emerge as promising tools to assist radiologists and clinicians in diagnosing and managing COVID-19 patients.

The aim of this study was to develop a deep learning-based clinical decision support system for the automatic diagnosis of COVID-19 on chest CT scans, that also included a segmentation algorithm to assess the extent of lung involvement⁠—by segmenting infectious lung opacities and calculating the volume of affected lung tissue—ultimately providing a measure of disease severity.

## Materials and methods

### Study design

The Imaging COVID-19 AI initiative was a large-scale collaborative effort to develop a generalisable deep-learning model for automatic classification and disease segmentation of chest CTs in COVID-19 suspected patients. For this purpose, a retrospective multicentre cohort study including 20 participant institutions from seven different European countries was conducted (S1 and S2 Tables). The study was approved by the Institutional Review Board of the Netherlands Cancer Institute (IRBd20-098). Study-specific informed consent was not required because of the retrospective nature of the study.

### Patient selection

Outpatient or hospitalised patients (≥18 years old) with suspected or known COVID-19 who underwent chest CT in secondary or tertiary referral centres were included in the study. The selection of patients was done through convenience sampling. The inclusion criteria for chest CT were as follows: (a) DICOM format, (b) with or without intravenous contrast, (c) volumetric series or axial reconstruction (recommended lung kernel), (e) slice thickness ≤ 3 mm (recommended ≤1.5 mm). For each collected CT study of the chest, an eligible series was manually selected by two radiologists (E.R., L.T.).

The diagnostic reference test for detection of SARS-CoV-2 was reverse RT-PCR. The sampling method was determined by the local centre, e.g., nasopharyngeal swab or bronchial lavage in selected cases.

### Imaging data

For this study, a dataset including CT scans routinely acquired from December 2019 through July 2020 was created. Imaging performed within seven days before or after the definitive diagnostic reference test were included. Each CT study was classified as “COVID-19”, “non-COVID-19 with another type of pulmonary infection”, or “non-COVID-19 with no imaging signs of infection”. The diagnostic criteria were based on collected laboratory test results and imaging findings (S3 Table). CT scans of patients that did not meet the diagnostic criteria were excluded for the classification model creation. Other relevant clinical information was also collected, including patient demographics (age, sex), hospitalisation status, and respiratory pathogen test results. Patients with missing clinical or imaging data were excluded.

For data collection, a secured web-based platform for manual uploading, or a local installation with PACS integration (Quibim SL, Valencia, Spain) was provided to the participating institutions. Pseudonymisation of the imaging data was performed locally, leaving any patient identifiable information on the client-side. Relevant clinical information was collected in a data transmittal form (DTF).

The dataset was split into a training, validation, and test sample (ratio 70:15:15) for both the classification/diagnosis and segmentation model creation. To assess the generalizability of the deep learning models, scans from different institutions were assigned to the training, validation, or test set (institution-level split).

### Data annotation

A custom annotation platform for medical imaging (Robovision, Ghent, Belgium) was developed to allow a collaborative decentralised annotation effort. The cloud-based platform incorporated image labelling software for 3D segmentation (ImFusion GmbH, München, Germany). In total, 34 annotators were recruited to perform the first read of the CT scans. The team of annotators included 29 radiologists and five radiology residents. The radiologists had an average of 16.6 years of experience, and 19 out of 29 were subspecialized in thoracic imaging. The annotators were assigned one or more batches of 50 CT scans. The annotations were reviewed by a dedicated team of two radiologists (E.R., L.T.) with 25 and 4 years of experience, respectively.

Each annotator received a video training with the intent of improving consistency of labelling [20]. The labelling consisted of manual segmentation of all infectious and non-infectious lung opacities and assigning text labels for classification on the image level. A comprehensive labelling system (S4 Table) was developed for this study in collaboration with an international initiative for a COVID-19 imaging database [21]. The annotator was assisted by a semi-automated brush tool for segmentation that used a 3D region-growing algorithm. After having labelled the first 994 CT scans, an automatic segmentation model was created to assist and speed up the further annotation process.

### Data preprocessing

For training the COVID-19 classification model, images were resized to 64x224x224 to speed up process and to allow GPU memory fitting. Window Level (WL) and Widow Width (WW) were set to -500 and 1500, respectively, to convert the image to lung window, therefore Hounsfield units (HU) were clipped to -1250 and 250, minimum and maximum values, respectively. Additionally, the pixel values were normalised to the range (0, 1) and a lung mask from a previous lung segmentation convolutional neural network (CNN) [22] was applied to set all voxels outside the lungs to 0.

For training the segmentation model each 2D image was resized to 320x320. The same window and normalisation were applied as for the classification task, but in this case, pixel information outside the lungs was not removed. Finally, to focus on the areas of interest, particularly the lung tissue, a specific pre-processing called balance contrast enhancement technique (BCET) was performed.

### COVID-19 classification model

**Fig 1:**
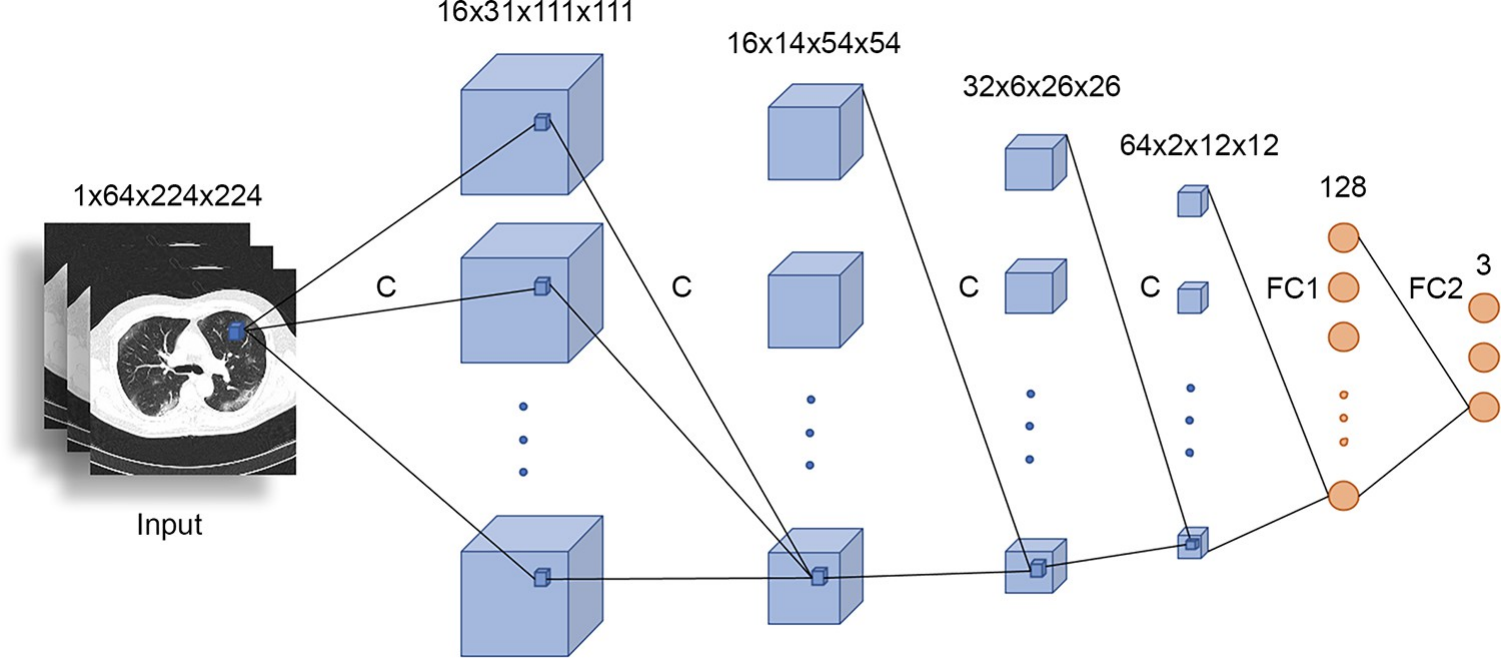
Custom 3D CNN architecture used for the classification model approach. C: 3x3x3 Conv + LeakyReLU + MaxPooling + Batch Normalization; FC1: 128 neuron fully connected layer; FC2: 3 neuron fully connected layer. CNN, convolutional neuronal network.

A deep learning model was developed that takes a whole CT scan as input data to perform a multi-class classification into the following categories: “COVID-19”, “Other type of pulmonary infection”, or “No imaging signs of infection”. The model consisted of a three-dimensional (3D) CNN whose architecture is shown in Fig 1. Briefly, the architecture consisted of four blocks including a 3D convolutional filter with LeakyReLU activation function, a 3D Max Pooling layer and a 3D Batch Normalization layer with 16, 16, 32, and 64 convolutional filters, respectively. Then, an adaptive Average Pooling 3D, a linear layer with 128 neurons using a LeakyReLU activation function and a Dropout layer of 30% and lastly, a linear layer with three neurons using a Softmax activation function. Therefore, the input of the network was a 3D volume, and the output was a vector of three dimensions with the probability associated to each of the three classes. Since the network architecture used was a custom one, the model parameters were randomly initialised using PyTorch inbuilt weight initialisation [23].

### Segmentation model

A deep learning segmentation model to assess the extent of lung involvement and disease severity was developed. The model provided a mask of infectious lung opacities.

The deep learning segmentation model used to extract the mask for the lung opacities was trained using a 2D approach. Each 3D volume was split into 2D slices and used as input for the model. The CNN architecture was a UNET-like architecture with a backbone Residual Network (ResNet-34), for both the encoder and decoder block. It was composed of 34 layers and each ResNet block was two layers deep. The prediction was made slice-wise and stacked into a 3D mask whose size matched the original one.

In addition, the lungs and lobes were segmented to calculate the percentage of affected lung tissue using a model previously developed for this purpose (Quibim Precision Platform 2.8) [22].

Finally, the segmentation tool included the calculation of a disease severity index score. The score was based on 25 points, 5 for each lung lobe, depending on the percentage of lung involvement ([0] no involvement, [1] less than 5% involvement, [2] 5%–25% involvement, [3] 26%–50% involvement, [4] 51%–75% involvement, and [5] 76%–100% involvement) as previously reported by Pan et al. [24]. The scores were added together to provide a total CT severity score ranging from 0 (no involvement) to 25 (maximum involvement).

### Training

To improve models’ generalization, different data augmentation techniques were randomly applied during the training process:

- Classification model: gaussian noise, with variance in the range of (0, 0.015), 3D rotations, elastic deformations using alpha in range of (0, 100) and sigma in range of (8,13), scaling in range of (0.9, 1.1), and mirroring on y and z axis.
- Segmentation model: random rotations in the range of (-20, 20) degrees, zooms in the range (0.95, 1.05) and lighting variations in the range (0%, 10%).

The COVID-19 classification model was trained during 200 epochs using a batch size of 10. The learning rate was initialised to 1e-04 and, after 50 epochs where the validation accuracy did not improve, the learning rate was reduced by a factor of 10.

The training process of the segmentation model consisted of 50 epochs divided in stages where the convolutional layers of the network were alternatively frozen and unfrozen and the learning rate was experimentally set in the range of (1e-07, 1e-02). In the frozen step, the learning rate was fixed stage-by-stage to the optimum value by plotting loss progression per epoch. In the unfrozen step, a range of values was chosen to be distributed along all the layers of the network, providing a lower value to the initial layers and a higher one to the deeper layers. In addition, the network was trained using three different input image sizes, from lower to higher, to learn the different patterns from more generic to more specific. The selected sizes were 128x128, 256x256 and 320x320 and the same routine was repeated for each one. The selected batch size was 64, except for the 320x320 resolution, which was halved to allow for memory fitting. The optimisation algorithm was Stochastic Gradient Descent (SGD), and the loss function was Binary Cross Entropy (BCE).

### Image analysis pipeline

An image analysis pipeline was built to process chest CTs and deliver a report with results to the physician. The pipeline started by performing data pre-processing steps. Then, the classification model provided a probability score for the three diagnostic classes. The following cut-off values for probabilities provided by the classification model were applied:

- If the probability of “No imaging signs of infection” > 0.45, the case was classified as “No imaging signs of infection”
- If the probability of “COVID-19” > 0.45, the case was classified as “COVID-19”. If not, the case was classified as “Other type of pulmonary infection”.

In parallel, the segmentation model provided a mask of infectious lung opacities, the percentage of affected lung tissue and a severity index score (“mild” [scores 1–5], “moderate” [scores 6–14] and “severe” [scores 15–25]). Finally, airways and vessels segmentation were performed to render a 3D image that was added to the report for visualisation purposes.

### Evaluation

For the COVID-19 classification model, the Area Under the Receiver Operating Characteristic curve (AUROC; both class-wise and average scores were obtained) was calculated. Sensitivity, specificity, positive-predictive value, and negative-predictive value of the likelihood of COVID-19 vs other cases were calculated. Performance metrics for the other binary classifications (“No imaging signs of infection” vs other cases and “non-COVID-19 with another type of pulmonary infection” vs other cases) were also obtained. To evaluate the performance of the segmentation model, DSC was calculated on all scans with at least 1,000 voxels in the ground truth segmentation. Because DSC is zero in cases without ground truth segmentations, this metric was only calculated for positive cases with infectious lung opacities. In addition, Pearson’s correlation coefficient between the predicted volume and the real one was determined.

## Results

### Patient and imaging characteristics

**Fig 2:**
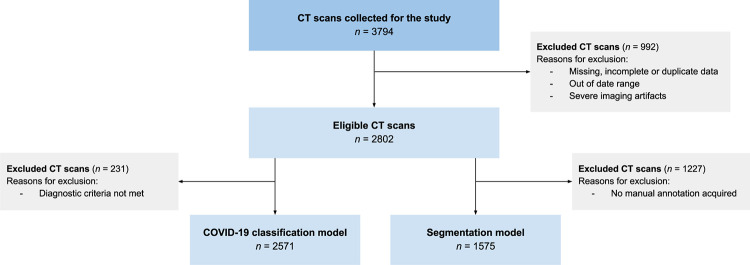
Data flowchart. COVID-19, coronavirus disease 2019; CT, computed tomography.

The data flow diagram is shown in Fig 2. A total of 2,802 CT scans were selected for the study, of which 2,571 met the inclusion criteria for the COVID-19 classification model creation and 1,575 (manually labelled) were used to develop and test the segmentation model. The patient demographics are listed in Table 1. The distribution of cases for the creation of both models according to the different classes established—“COVID-19”, “Other type of pulmonary infection”, or “No imaging signs of infection”—is shown in Table 2. For the COVID-19 classification model, the training and validation dataset (*n* = 2,097 CTs) consisted of 1,979 unique patients (mean age [SD] = 62.1 [16.3] years, male/female [M/F] ratio 1.3:1). The test dataset (*n* = 474 CTs) consisted of 474 unique patients (mean age 65.5 [16.0] years, M/F ratio 1.4:1). For the segmentation model, the training and validation dataset (*n* = 1,334 CTs) consisted of 1,307 unique patients (mean age [SD] = 63.1 [16.6] years, M/F ratio 1.2:1). The test dataset (*n* = 241 CTs) consisted of 241 unique patients (mean age [SD] = 64.7 [15.3] years, M/F ratio 1.9:1).

**Table 1:** Patient demographics.

Eligible CT scans (*n*)	2,802
Unique patients (*n*)	2,667
Mean age ± SD (years)	64.6 ± 16.2
Male/female ratio	1.3:1
Hospitalisation status^a^ (*n*)	
Inpatient	1,121 (71.8%)
Inpatient—ICU	144 (9.2%)
Outpatient	441 (28.2%)

CT, computed tomography; ICU, intensive care unit; SD, standard deviation.

^a^Patient hospitalisation status was known for 1562 (55.7%) CT scans.

**Table 2:** Distribution of diagnostic classes for both COVID-19 classification and segmentation models.

	Training set *n* (%)		Validation set *n* (%)		Test set *n* (%)		Total *n* (%)	
	Classification	Segmentation	Classification	Segmentation	Classification	Segmentation	Classification	Segmentation
**COVID-19**	923 (54.5)	544 (50.2)	218 (54.0)	126 (50.4)	278 (58.6)	154 (63.9)	1,419 (55.2)	824 (52.3)
**Other type of pulmonary infection**	265 (15.7)	189 (17.4)	38 (9.4)	36 (14.4)	63 (13.3)	23 (9.5)	366 (14.2)	248 (15.7)
**No imaging signs of infection**	505 (29.8)	351 (32.4)	148 (36.6)	88 (35.2)	133 (28.1)	64 (26.6)	786 (30.6)	503 (31.9)
**TOTAL**	**1,693**	**1,084**	**404**	**250**	**474**	**241**	**2,571**	**1,575**
**Included institutions**	**11**		**3**		**6**		**20**	

COVID-19, coronavirus disease 2019

The data distribution per institution for the classification and segmentation model creation is shown in S1 Fig. Imaging was acquired on CT scanners from four manufacturers (GE, Philips, Siemens, and Toshiba), including 27 different vendor models of which the details are available in S5 Table. The variation in slice thickness ranged from 0.63 to 3.0 mm; average 1.6 mm.

### COVID-19 classification model performance

**Fig 3:**
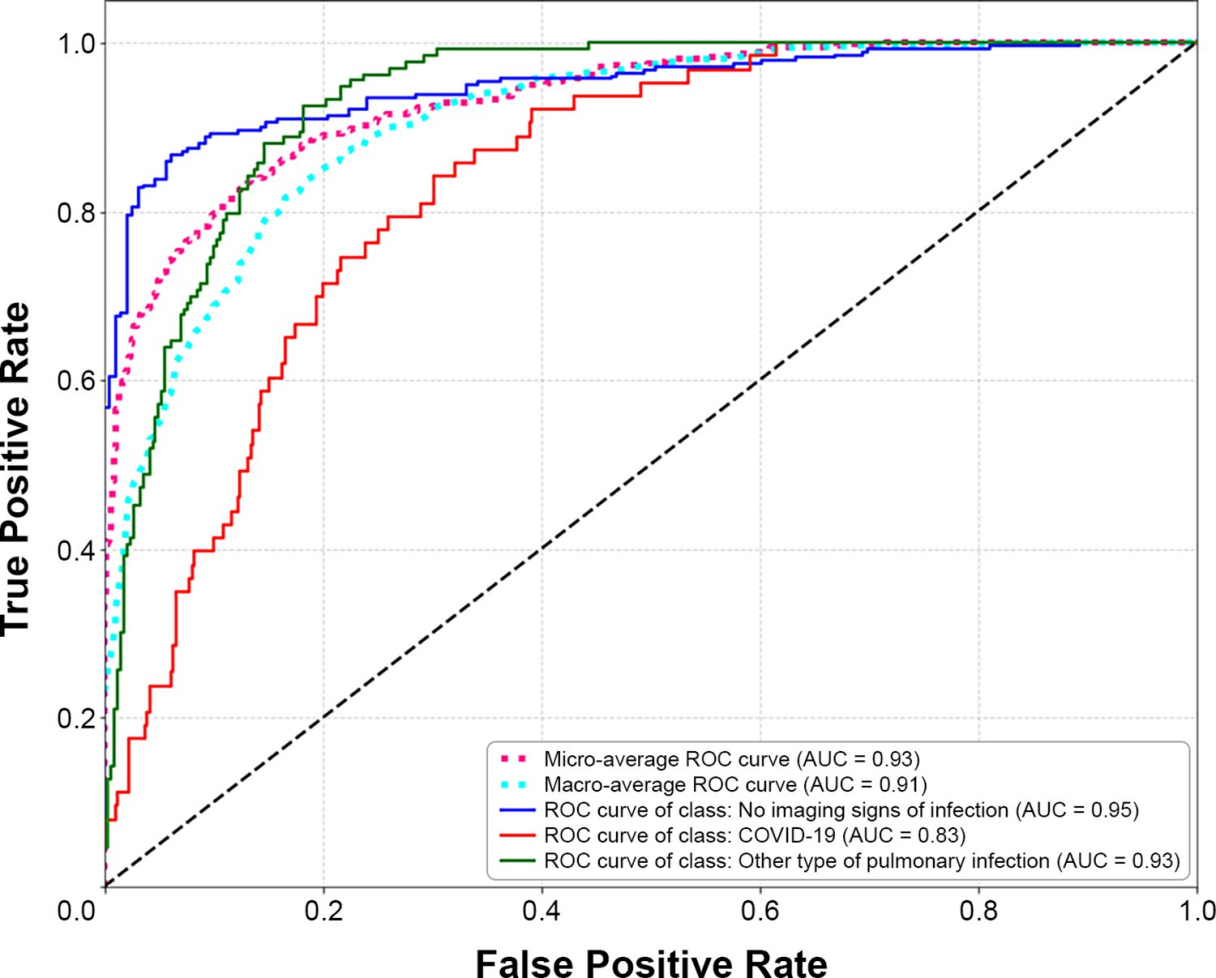
Receiver operating characteristic (ROC) curve for the COVID-19 classification model. AUC, area under the ROC curve.

**Fig 4:**
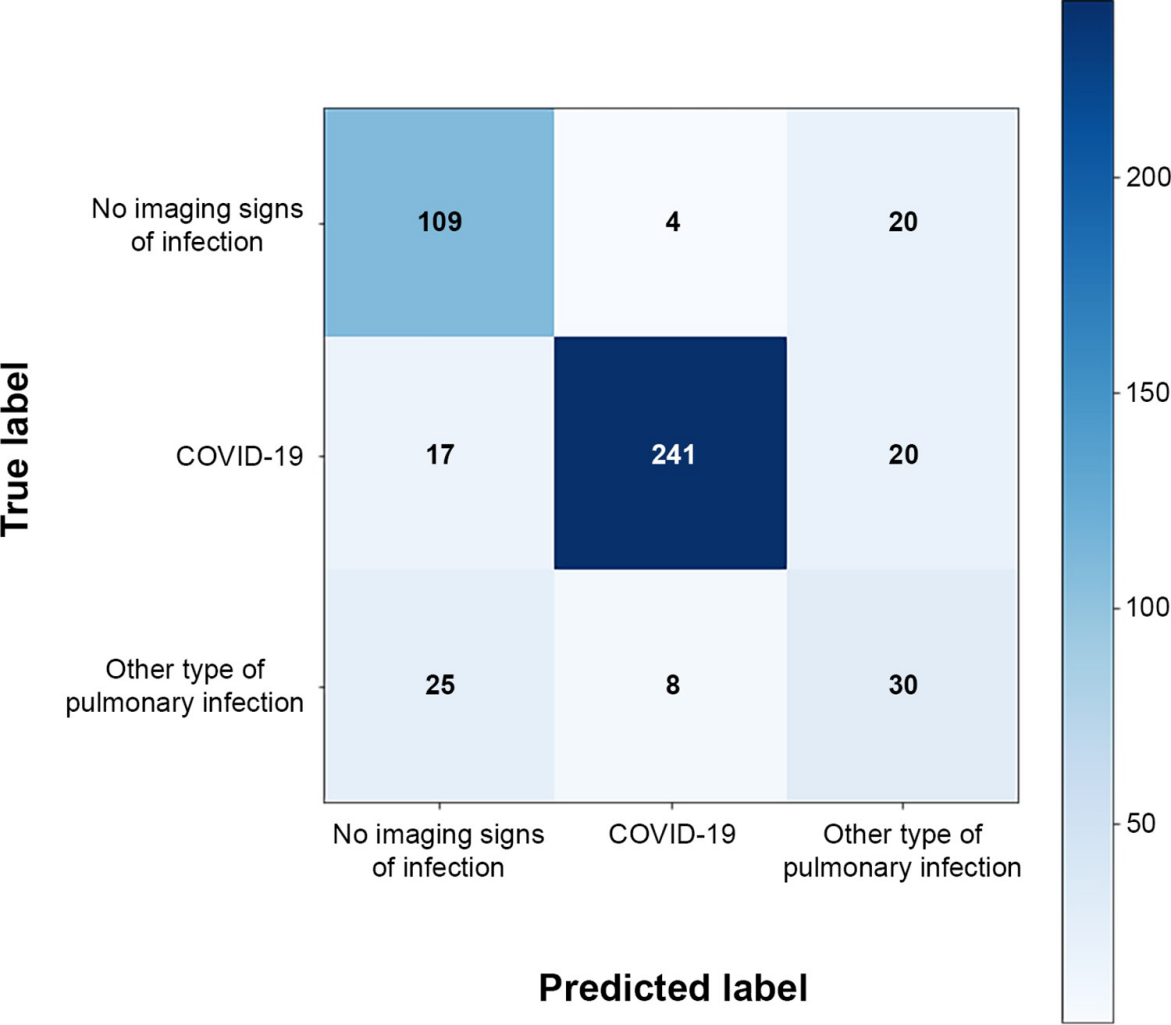
Confusion matrix for the COVID-19 classification model. COVID-19, coronavirus disease 2019.

The performance of the COVID-19 classification model on the test dataset is shown in Fig 3. As observed, on average, the multiclassification model yielded high AUC values (micro-average AUC = 0.93; macro-average AUC = 0.91), with good sensitivity (87%), specificity (94%), and accuracy (90%), and with good positive and negative predictive values (95% and 83%, respectively) for the likelihood of COVID-19 vs other cases (Table 3). Performance metrics for the other binary classifications are shown in S6 Table. The confusion matrix for the model is shown in Fig 4. Importantly, no false positives for COVID-19 were detected if the probability score was 0.75 or higher.

**Table 3:** Performance metrics for the COVID-19 classification model (COVID-19 vs other cases).

Performance metrics	COVID-19 classification model
AUC	0.83
Sensitivity	0.87
Specificity	0.94
Accuracy	0.90
PPV	0.95
NPV	0.83

AUC, area under the curve; COVID-19, coronavirus disease 2019; NPV, negative predictive value; PPV, positive predictive value

### Segmentation model performance

The final test dataset for assessing the segmentation performance was determined by a histogram-based threshold of CT scans with more than 1,000 positive voxels, thereby including 167 scans, and excluding 74 scans. The performance metrics for segmentation are shown in Table 4. The model provided faithful visual results, within lung contours, and sensitive to small ground-glass opacities.

**Table 4:** Segmentation model performance metrics.

Metric	Statistics	Value
Total DSC	Mean	0.59
	SD	0.20
Pearson correlation	Correlation coefficient	0.92
	Two tailed p-value	< 0.001

DSC, Dice similarity coefficient; SD, standard deviation.

### Deployment and output results

**Fig 5:**
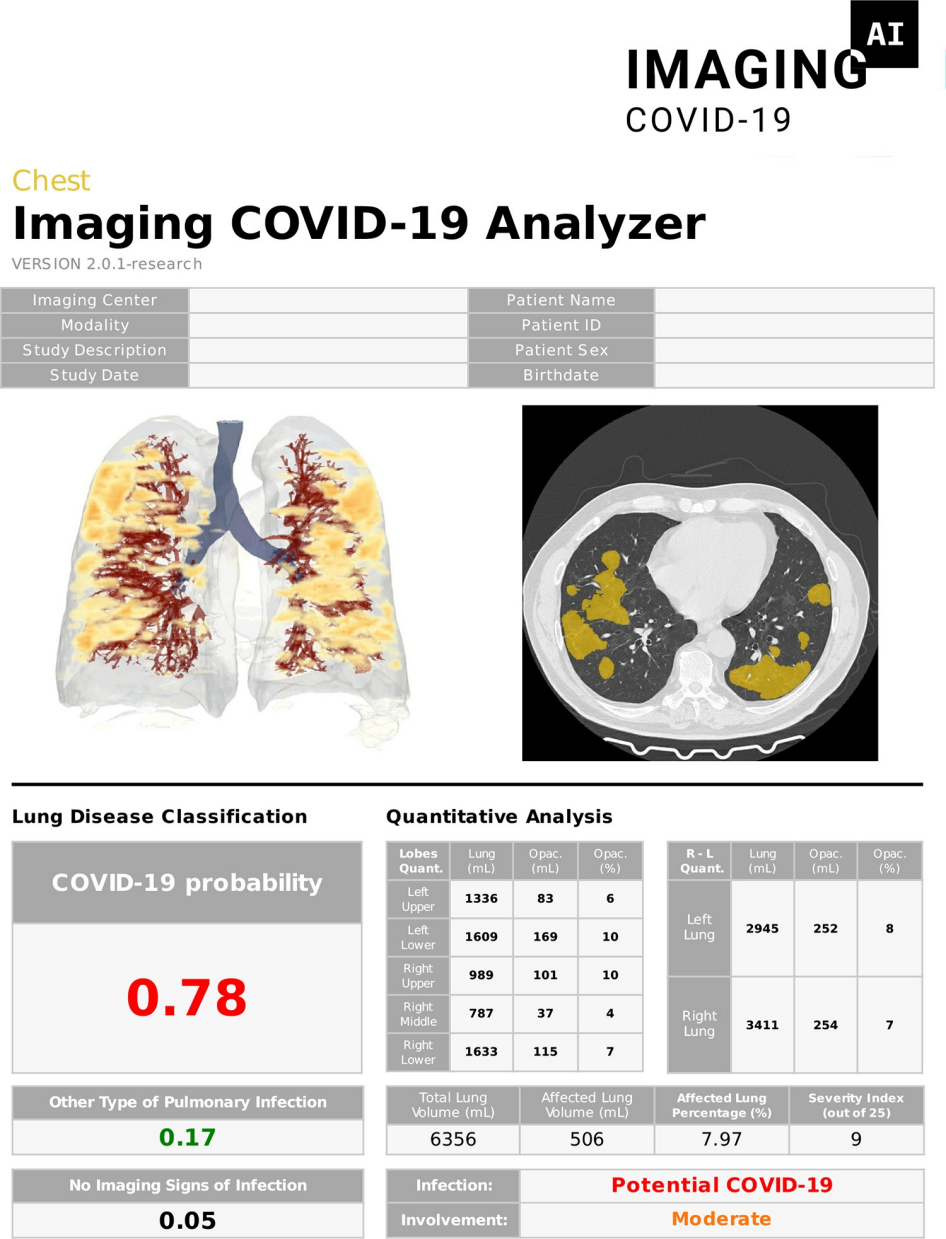
Structured report with analysis results. A 3D reconstruction of the lungs is generated, together with the most affected transverse CT slice and segmentation masks of the lung opacities. The report includes disease probabilities and quantitative analysis results. COVID-19, coronavirus disease 2019; CT, computed tomography.

An example of the output returned to the user is shown in Fig 5, mentioning both the diagnostic classification and segmentation results. The developed models and image analysis pipeline were containerized using Docker technology [25]. The resulting application was made accessible in a freely available online platform for research purposes (https://imagingcovid19.quibim.com/). The web-based platform was used by more than 300 users from four different continents, who executed more than 7,000 analyses. In addition, the application was made available through a local installation with (research) picture archiving and communication system (PACS) integration. The on-premise installation allowed automatic analysis of eligible CT scans after acquisition and was installed in more than 10 institutions.

## Discussion

We developed and evaluated an automated deep learning-based application for the diagnosis of COVID-19 on chest CT images. In addition, the tool performed segmentation of infectious lung opacities, enabling the calculation of the extent of lung involvement, as well as the prediction of COVID-19 disease severity. As a result of the image analysis pipeline using both models, a complete and visual report can be delivered that can assist clinicians in the decision-making process of suspected and known COVID-19 patients.

Our results demonstrated excellent performance (micro-average AUC = 0.93; macro-average AUC = 0.91) in the classification task, by differentiating COVID-19 versus other types of pulmonary infection or no imaging signs of infection. This corresponds to what was reported earlier. In a recently published systematic review [26], in which the available data on the AI-assisted CT-scan prediction accuracy for COVID-19 were reviewed, 18 studies developing AI models based on CNN were identified. These models showed excellent ability to discriminate COVID-19 and non-COVID pneumonia with an accuracy of 70% to 99.9%, sensitivity of 73% to 100%, specificity of 25% to 100%, and AUC of 0.73 to 1. The values produced by our model for these performance metrics for the classification of COVID-19 cases versus other cases were also within those ranges, with an accuracy of 90%, a sensitivity of 87%, a specificity of 94% and an AUC of 0.83, with micro- and macro-averaged AUC values of 0.93 and 0.91 for the multi-class classification, respectively. On the other hand, in our test dataset, there were no false positives for COVID-19 if the probability score was 0.75 or higher. The tool has the potential to support the radiologist during image interpretation, as distinguishing between COVID-19 and other pneumonias may be challenging. Indeed, Bai et al. [27] found that AI assisted interpretation improved the radiologists’ sensitivity and specificity in discriminating COVID-19 from other types of pneumonia on chest CT. Likewise, Zhang et al. [28] reported that the performance of their AI system was overall superior to that of junior radiologists and comparable to mid-senior radiologists.

Importantly, in the majority of studies published so far, deep learning models were developed and validated with newly created datasets limited in size [29, 30], or including CT scans exclusively or almost exclusively acquired in China [28, 31]. To the best of our knowledge, our study is the only one to date using a newly created multicentre dataset of more than 2,800 CT scans acquired from 20 institutions located in 7 different European countries during the first wave of the pandemic. The performance and generalisability were assessed on external data containing 483 patients from six institutions, which strengthens our conclusions.

It is recognised that CT imaging can be used to assess the severity of COVID-19 [32–36]. However, interpretation of disease extent may be subject to interobserver variability. Automated calculation of parenchymal involvement can therefore provide a fast and reproducible way to assess the disease severity and help with prognosis. In this work, we also developed a deep learning model to assess the extent of lung involvement by segmenting COVID‐19 infection on CT images, ultimately providing information regarding the disease severity. In our study, the segmentation performance (DSC = 0.59) was moderate, probably because of the diversity of the external test dataset that was used. Although several studies have reported higher DSC values, it is worth mentioning that most of them were single centre studies, were performed with small sample sizes or did not assess model performance on an external test dataset [37–39]. Our results are similar to those obtained in another multicentre study carried out with a large cohort from China, in which a mean DSC of 0.587 for lesion segmentation was reported [28]. It is also important to note the remarkable annotation effort in this study, which brought together a large group of annotators consisting of 29 radiologists with an average of 16.6 years of experience. Additionally, data annotations were reviewed by two dedicated radiologists to achieve accurate and consistent labels.

In a recent systematic review, Roberts et al. [40] reported a high prevalence of deficiencies in methodology of AI studies for detection and prognostication of COVID-19 on imaging. These limitations include poor-quality data, low reproducibility, and biases in study design. In this study, we tried to overcome some of those issues. First, as discussed earlier, data were collected from several European institutions, both academic and non-academic, creating a diverse dataset with different scanning protocols and image qualities. Secondly, data collection, dataset split, data pre-processing, training approach, model creation, and performance metrics were reported in detail to increase the reproducibility of our study. Finally, control patients were selected during the same time period as COVID-19 patients to avoid discrepancies in imaging protocols that could bias the classification task.

However, this study also has some limitations. First, patients were selected by convenience sampling during a period of high incidence rates of COVID-19 in Europe. This resulted in a class imbalance with overrepresentation of COVID-19 patients versus other causes of pneumonia. Secondly, RT-PCR was used as a reference standard. The sensitivity of RT-PCR is imperfect, which can result in false negative cases. Furthermore, not all patients in the non-COVID-19 pneumonia group had a proven respiratory disease. Finally, the segmentation performance was compared to manual segmentation, which is known to have a high interrater variability.

## Conclusion

As a result of the Imaging COVID-19 AI initiative, a large-scale collaborative effort involving 20 institutions from seven countries in Europe, a generalizable deep learning-based application was developed that performed automated COVID-19 diagnosis and allowed to assess the extent of lung involvement and disease severity. We believe that our system could become an efficient first or concurrent reading tool to assist clinicians and radiologists, especially during outbreak periods, in which a significant demand for diagnostic expertise is required and in which molecular testing may be time-consuming and/or limited (e.g., remote areas). The assistance provided by this automatic tool may improve patient’s triaging and management without adding much cost, by reducing waiting time and shortening diagnostic workflow time, subsequently allowing a more efficient and quicker response in an emergency situation. In the future, the clinical impact of the developed application on the diagnostic accuracy and efficiency of the radiologist will need to be further investigated.

## Supporting information

S1 Fig Data distribution per institution. For the creation of (A) COVID-19 classification and (B) segmentation models. COVID-19, coronavirus disease 2019; CT, computed tomography.
(TIF)

S1 Table Participant institutions and data contribution. (DOCX)

S2 Table Participating institutions per country. (DOCX)

S3 Table Diagnostic criteria for classification of CT scans. (DOCX)

S4 Table Data labelling system. (DOCX)

S5 Table Distribution of CT scanner models. (DOCX)

S6 Table Performance metrics for the binary classifications performed by the COVID-19 classification model. (DOCX)
