# Supplementary figures and images for: A deep learning-based application for COVID-19 diagnosis on CT: The Imaging COVID-19 AI initiative
Source: PLoS One. 2023 May 2;18(5):e0285121. doi: 10.1371/journal.pone.0285121 (PMC10153726; doi:10.1371/journal.pone.0285121)

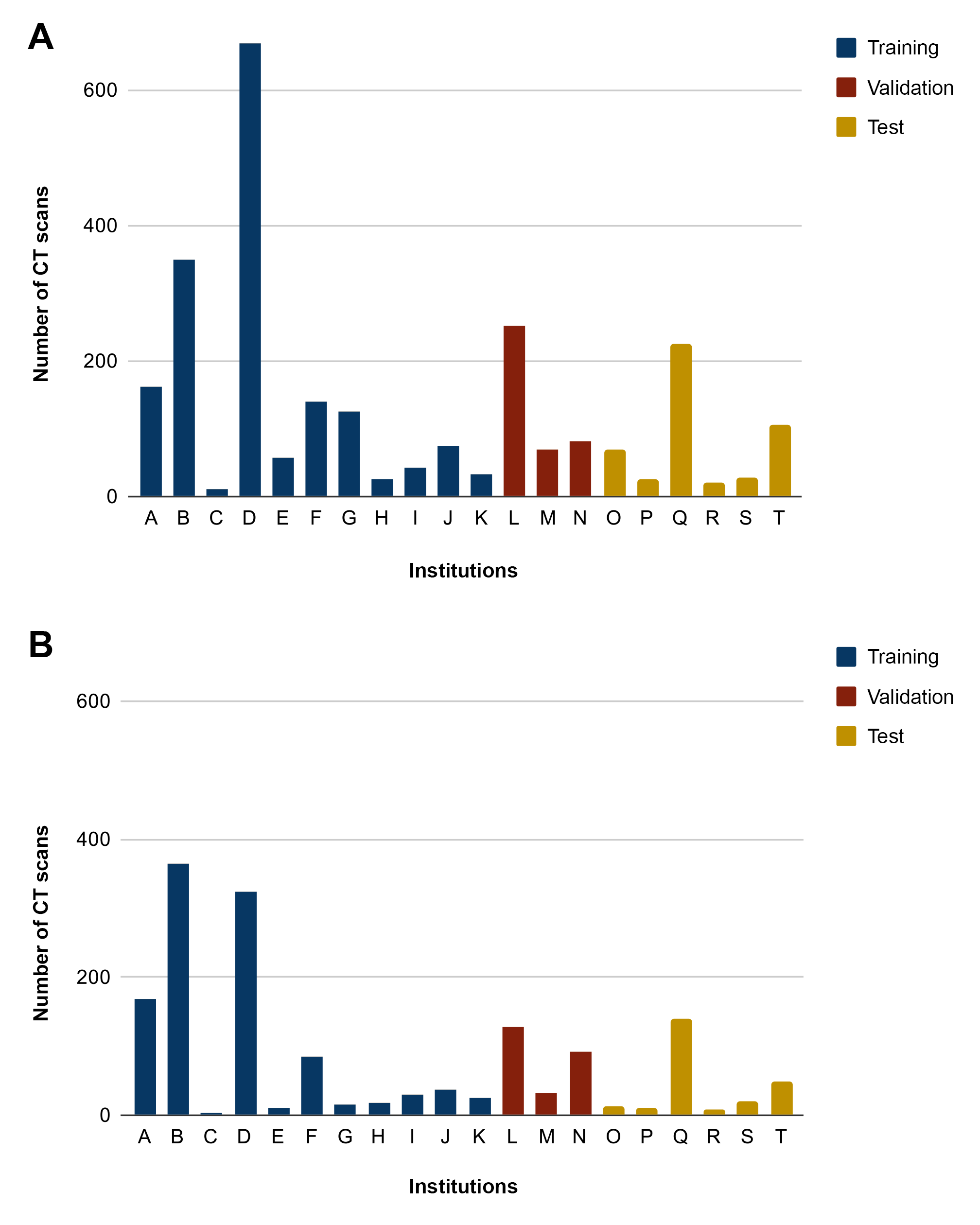

Supplement: S1 Fig — For the creation of (A) COVID-19 classification and (B) segmentation models. COVID-19, coronavirus disease 2019; CT, computed tomography. (TIF) [file pone.0285121.s001.tif]
