# Supplementary material for: A deep learning-based application for COVID-19 diagnosis on CT: The Imaging COVID-19 AI initiative
Source: PLoS One. 2023 May 2;18(5):e0285121. doi: 10.1371/journal.pone.0285121 (PMC10153726; doi:10.1371/journal.pone.0285121)
Supplement: S1 Table — (DOCX) [file pone.0285121.s002.docx]

**S1 Table. Participant institutions and data contribution.**

|  | **Institution** | **Location** | ***n* CT scans** | |
| --- | --- | --- | --- | --- |
| A | OLV Hospital | Aalst, Belgium | 186 | |
| B | Antwerp University Hospital | Antwerp, Belgium | 406 | |
| C | Freiburg University Medical Center | Freiburg, Germany | 11 | |
| D | CHU de Liège | Liège, Belgium | 728 | |
| E | Hôpitaux Robert Schuman | Luxembourg, Luxembourg | 63 | |
| F | Laurentius Hospital | Roermond, Netherlands | 150 | |
| G | Ikazia Hospital | Rotterdam, Netherlands | 129 | |
| H | Maasstad Hospital | Rotterdam, Netherlands | 28 | |
| I | Università Campus Bio-Medico di Roma | Rome, Italy | 51 | |
| J | Hospital Universitari Mútua Terrassa | Terrassa, Spain | 84 | |
| K | Alvaro Cunqueiro Hospital | Vigo, Spain | 42 | |
| L | AZ Sint-Blasius | Dendermonde, Belgium | 275 | |
| M | Zuyderland Medical Center | Heerlen, Netherlands | 71 | |
| N | AOU Città della Salute e della Scienza | Turin, Italy | 95 | |
| O | Noordwest Ziekenhuisgroep | Alkmaar, Netherlands | 72 | |
| P | GAONA St. Savvas | Athens, Greece | 25 | |
| Q | Amphia Hospital | Breda, Netherlands | 226 | |
| R | Europe Hospitals | Brussels, Belgium | 24 | |
| S | Hospital Universitario y Politécnico La Fe | Valencia, Spain | 28 | |
| T | AZ West | Veurne, Belgium | 108 | |
| CT, computed tomography. | | | |  |
