# Supplementary material for: A deep learning-based application for COVID-19 diagnosis on CT: The Imaging COVID-19 AI initiative
Source: PLoS One. 2023 May 2;18(5):e0285121. doi: 10.1371/journal.pone.0285121 (PMC10153726; doi:10.1371/journal.pone.0285121)
Supplement: S2 Table — (DOCX) [file pone.0285121.s003.docx]

**S2 Table. Participating institutions per country.**

| **Country** | **Number of institutions** |
| --- | --- |
| Belgium | 6 |
| Germany | 1 |
| Greece | 1 |
| Italy | 2 |
| Luxembourg | 1 |
| The Netherlands | 6 |
| Spain | 3 |
| **Total** | **20** |
