# Supplementary material for: A deep learning-based application for COVID-19 diagnosis on CT: The Imaging COVID-19 AI initiative
Source: PLoS One. 2023 May 2;18(5):e0285121. doi: 10.1371/journal.pone.0285121 (PMC10153726; doi:10.1371/journal.pone.0285121)
Supplement: S3 Table — (DOCX) [file pone.0285121.s004.docx]

**S3 Table. Diagnostic criteria for classification of CT scans.**

| **Diagnosis** | **Criteria** |
| --- | --- |
| COVID-19 | Positive RT-PCR test for SARS‐CoV‐2 |
| Non-COVID-19 with other type of pulmonary infection | Negative RT-PCR test for SARS‐CoV‐2  **and**  positive laboratory test for other respiratory pathogen or imaging signs of infection |
| Non-COVID-19 with no imaging signs of infection | Negative RT-PCR test for SARS‐CoV‐2  **and**  no imaging signs of infection |
| COVID-19, coronavirus disease 2019; CT, computed tomography; RT-PCR, reverse transcription polymerase chain reaction; SARS-CoV-2, severe acute respiratory syndrome coronavirus 2. | |
