# Supplementary material for: A deep learning-based application for COVID-19 diagnosis on CT: The Imaging COVID-19 AI initiative
Source: PLoS One. 2023 May 2;18(5):e0285121. doi: 10.1371/journal.pone.0285121 (PMC10153726; doi:10.1371/journal.pone.0285121)
Supplement: S4 Table — (DOCX) [file pone.0285121.s005.docx]

**S4 Table. Data labelling system.**

| **Segmentation labels (voxel-level)** |
| --- |
| Segmentation of imaging abnormalities |
| Infectious lung opacity (ground-glass opacity, consolidation) |
| Infectious tree-in-bud pattern / micronodules |
| Infectious cavity |
| Non-infectious nodule / mass |
| Non-infectious atelectasis |
| Other non-infectious opacity |
| **Classification labels (image-level)** |
| Specific and additional imaging findings |
| Halo sign |
| Reversed halo sign |
| Reticular pattern without parenchymal opacification |
| Perilesional vessel enlargement |
| Bronchial wall thickening |
| Bronchiectasis/bronchiolectasis |
| Subpleural curvilinear line |
| Pleural effusion |
| Pleural thickening |
| Pneumothorax |
| Pericardial effusion |
| Type of lung disease |
| Normal lungs |
| Infectious lung disease |
| Pulmonary emphysema |
| Oncologic lung disease |
| Non-infectious inflammatory lung disease |
| Non-infectious interstitial lung disease |
| Fibrotic lung disease |
| Other type of lung disease |
| RSNA COVID-19 classification [1] |
| Typical appearance of COVID-19 |
| Indeterminate for COVID-19 |
| Atypical for COVID-19 |
| Negative for pneumonia |
| Quality control |
| Adequate |
| Inadequate – Severe motion or breathing artifact |
| Inadequate – Insufficient inspiration |
| Inadequate – Very low resolution |
| Inadequate – Incomplete lungs |
| Inadequate – Wrong body part or modality |
| Intravenous contrast |
| Without IV contrast |
| With IV contrast |
| Presence of support apparatus |
| Endotracheal tube |
| Central venous/arterial line |
| Nasogastric tube |
| Sternotomy wires |
| Pacemaker |
| Other |
| COVID-19, coronavirus disease 2019; IV, intravenous; RSNA, Radiological Society of North America. |

**References**

1. Simpson S, Kay FU, Abbara S, Bhalla S, Chung JH, Chung M, et al. Radiological Society of North America Expert Consensus Document on Reporting Chest CT Findings Related to COVID-19: Endorsed by the Society of Thoracic Radiology, the American College of Radiology, and RSNA. Radiol Cardiothorac Imaging. 2020;2(2):e200152.
