# Supplementary material for: A deep learning-based application for COVID-19 diagnosis on CT: The Imaging COVID-19 AI initiative
Source: PLoS One. 2023 May 2;18(5):e0285121. doi: 10.1371/journal.pone.0285121 (PMC10153726; doi:10.1371/journal.pone.0285121)
Supplement: S5 Table — (DOCX) [file pone.0285121.s006.docx]

**S5 Table. Distribution of CT scanner models.**

| **Manufacturer** | ***n* CT scans** | **Model** | ***n* CT scans** |
| --- | --- | --- | --- |
| GE | 1215 (43.4%) | BrightSpeed | 302 |
|  |  | Discovery CT750 HD | 14 |
|  |  | Discovery MI | 1 |
|  |  | Discovery RT | 360 |
|  |  | LightSpeed 16 | 1 |
|  |  | LightSpeed VCT | 88 |
|  |  | Optima CT660 | 6 |
|  |  | Revolution CT | 210 |
|  |  | Revolution EVO | 121 |
|  |  | Revolution HD | 6 |
|  |  | Unknown | 106 |
| Philips | 385 (13.7%) | Brilliance 64 | 3 |
|  |  | iCT256 | 58 |
|  |  | Incisive CT | 44 |
|  |  | Ingenuity Core | 4 |
|  |  | Ingenuity CT | 33 |
|  |  | MX 16-slice | 243 |
| Siemens | 1170 (41.8%) | Biograph 64 | 12 |
|  |  | Emotion 16 | 125 |
|  |  | Sensation 64 | 2 |
|  |  | SOMATOM Definition AS | 269 |
|  |  | SOMATOM Definition Edge | 8 |
|  |  | SOMATOM Definition Flash | 242 |
|  |  | SOMATOM EdPlus | 277 |
|  |  | SOMATOM Force | 56 |
|  |  | Symbia Intevo 16 | 179 |
| Toshiba | 32 (1.1%) | Aquilion | 24 |
|  |  | Aquilion Prime | 5 |
|  |  | Unknown | 3 |
| CT, computed tomography. | | | |
