# Supplementary material for: A deep learning-based application for COVID-19 diagnosis on CT: The Imaging COVID-19 AI initiative
Source: PLoS One. 2023 May 2;18(5):e0285121. doi: 10.1371/journal.pone.0285121 (PMC10153726; doi:10.1371/journal.pone.0285121)
Supplement: S6 Table — (DOCX) [file pone.0285121.s007.docx]

**S6 Table. Performance metrics for the binary classifications performed by the COVID-19 classification model.**

| **Performance metrics** | **No imaging signs of infection** | **Other type of pulmonary infection** |
| --- | --- | --- |
| AUC | 0.95 | 0.93 |
| Accuracy | 0.86 | 0.85 |
| Sensitivity | 0.82 | 0.48 |
| Specificity | 0.88 | 0.90 |
| PPV | 0.72 | 0.43 |
| NPV | 0.93 | 0.92 |
| AUC, area under the curve; COVID-19, coronavirus disease 2019; NPV, negative predictive value; PPV, positive predictive value | | |
